# An Update Summary on the Learning Sciences Within an Ophthalmic Context

**DOI:** 10.7759/cureus.53288

**Published:** 2024-01-31

**Authors:** Maryam Mushtaq, Yusuf Mushtaq, Aishwarya Khanna, Ahmed Javed

**Affiliations:** 1 College of Medicine, Luton and Dunstable Hospital, Bedfordshire Hospitals NHS Foundation Trust, Luton, GBR; 2 Ophthalmology, Nottingham University Hospitals NHS Trust, Nottingham, GBR; 3 Ophthalmology, Royal Derby Hospital, Derby, GBR; 4 Vitreoretinal, Birmingham & Midland Eye Centre, Birmingham, GBR

**Keywords:** effective decision-making skills, decision making process, process & performance improvement, cognitive abilities, diagnostic errors, problem-solving, diagnostic reasoning, critical reasoning, ophthalmology, evidenced based medical education

## Abstract

Clinical reasoning, specifically diagnostic decision-making, has been a subject of fragmented literature since the 1970s, marked by diverse theories and conflicting perspectives. This article reviews the latest evidence in medical education, drawing from scientific literature, to offer ophthalmologists insights into optimal strategies for personal learning and the education of others. It explores the historical development of clinical reasoning theories, emphasising the challenges in understanding how doctors formulate diagnoses. The importance of clinical reasoning is underscored by its role in making accurate diagnoses and preventing diagnostic errors. The article delves into the dual process theory, distinguishing between type 1 and type 2 thinking and their implications for clinical decision-making. Cognitive load theory is introduced as a crucial aspect, highlighting the limited capacity of working memory and its impact on the diagnostic process.

The zone of proximal development (ZPD) is explored as a framework for optimal learning environments, emphasising the importance of scaffolding and deliberate practice in skill development. The article discusses semantic competence, mental representation, and the interplay of different memory stores-semantic, episodic, and procedural-in enhancing diagnostic proficiency. Self-regulated learning (SRL) is introduced as a student-centric approach, emphasising goal setting, metacognition, and continuous improvement. Practical advice is provided for minimising cognitive errors in clinical reasoning, applying dual process theory, and considering cognitive load theory in teaching. The relevance of deliberate practice in ophthalmology, especially in a rapidly evolving field, is emphasised for continuous learning and staying updated with advancements.

The article concludes by highlighting the importance of clinical supervisors in recognising and supporting trainees' self-regulated learning and understanding the principles of various teaching and learning theories. Ultimately, a profound comprehension of the science behind clinical reasoning is deemed fundamental for ophthalmologists to deliver high-quality, evidence-based care and foster critical thinking skills in the dynamic landscape of ophthalmology.

## Introduction and background

The subject of clinical reasoning has been a fragmented literature since the 1970s. Clinical reasoning, or diagnostic decision-making, has attempted to be understood with many theories and heterogeneous answers that span disagreements over the last four decades [1].

This narrative review seeks to summarise the latest evidence from medical education and learning of relevant scientific literature to provide ophthalmologists insight into how best to foster their own learning and the development and education of others. We employed a keyword-based methodology, entering terms such as 'type 1 thinking,' 'memory store types,' and 'zone of proximal development (ZPD)' into databases to retrieve relevant information for analysis and exploration.

A brief history of clinical reasoning

The early era of clinical reasoning literature aimed to capture the process by which doctors come to a diagnosis. To put simply, if one could understand that process as a skill, it could then be taught to novices to facilitate faster and more efficient learning. The problem that was quickly encountered was trying to determine exactly how doctors formulate diagnoses. That sparked much debate in the various disciplines of psychology, education, and medicine [1]. Various attempts have been used to try and elucidate the process of diagnostic reasoning. These include methods such as ‘think aloud’ where the clinician verbally described their thoughts and processes while being questioned about a patient interaction [2]. This is a commonly used assessment method today in objective structured clinical examinations (OSCE) to determine how trainee healthcare professionals come to a decision. Finally, there are novel methods, such as script concordance, to determine what factors make a diagnosis more likely in the clinician’s mind. In a scoping review by Daniel et al. in 2019 [3], the existing menu of clinical reasoning assessments can be categorised into three types: non-workplace-based assessments (e.g., multiple-choice questions and extended matching questions); assessments in simulated clinical environments (OSCEs); and workplace-based assessments (e.g., direct observations and oral case presentations).

Why is clinical reasoning important?

The early intentions of the educationalist to identify the process of thought were to be able to impart that process on to naive learners and therefore allow them to learn the specific skill of clinical, diagnostic reasoning [4]. Knowledge of specific terms, clinical signs and presentations are of central importance to an accurate diagnosis, and previous experience of these signs and symptoms is essential. In truth, the process of how novices and experts make a diagnosis is largely the same, experts are just more accurate. The process of how this happens has become more agreed upon with the development of the dual process theory [5]. However, the importance of clinical reasoning lies in the ability to make accurate diagnoses and prevent diagnostic errors. In the field of ophthalmology, professionals frequently contend with the intricacies of clinical reasoning and diagnostic decision-making. This article, therefore, aims to highlight the challenges faced by these practitioners in developing the correct diagnoses and preventing errors. Ultimately, the employment of effective clinical reasoning in ophthalmology contributes greatly to improved patient outcomes. Accurate diagnoses and tailored treatment plans lead to better management of eye conditions, thereby preserving or enhancing patients' vision and overall eye health. Furthermore, effective clinical reasoning in ophthalmology necessitates a dedication from ophthalmologists to continuous learning and staying abreast of the latest advancements. This ongoing education ensures that doctors remain adept at applying the best practices and incorporating advancements into their patient care.

## Review

Clinical reasoning and diagnostic error

Diagnostic errors in internal medicine have been studied by Graber et al [6]. The study aimed to determine the relative contribution of system-related and cognitive components to diagnostic error. It was deemed that cognitive error was the most significant and common cause of diagnostic error and subsequent patient harm. Cognitive errors entail situations where all essential information for an accurate diagnosis is available, yet an incorrect diagnosis is reached due to lapses in reasoning or judgement. While the study acknowledged the role of systematic errors, machine failures, and other types of errors in contributing to diagnostic inaccuracies and patient harm, it underscored the heightened impact of cognitive errors on morbidity. Therefore, in the realm of ophthalmology, enhancing these cognitive abilities through regular teaching, supervision and wider reading is crucial for augmenting patient safety and mitigating diagnostic errors.

Dual process theory

If that is the case, how can clinicians help themselves and others learn? Firstly, there are several new and important theories on how they make diagnoses that have become more universally agreed. The first of these to understand is the dual process theory by Kahneman [7]. In his book ‘Thinking, Fast and Slow’ he discusses the role of type 1 and type 2 thinking and this is important to understand and contextualise from an ophthalmologist’s point of view.

Type 1 Thinking

Type 1 thinking is an evolutionarily preserved system of reflex-like thinking. It preserves cognitive function and allows rapid access to long-term memory without imposing a heavy burden on working memory. Therefore, when a clinician of experience recognises a constellation of signs and symptoms, they immediately access their type 1 thinking. This is thought to be associated with their long-term memory, termed semantic memory [8,9]. This accesses a network of knowledge bundles existing in their semantic memory of terms, diagnoses, signs and all learned information that is interrelated resulting in an instant diagnosis. Excellent clinicians can then further verify this with confirmatory signs and tests from examination findings and do a pause check to ensure everything fits and they have answered the question, ‘What else could it be?’ Prior to providing their diagnoses and management plan [10,11].

There are significant criticisms of this process as it is seen as a form of pattern recognition and susceptible to the invasion of biases. This includes satisficing, or premature closure, whereby the optimal solution is not achieved as other potential contributing factors are not addressed. For example, when dealing with contact lens wearers presenting with a red eye in the ophthalmology department, the initial inclination might be to consider a diagnosis of a corneal ulcer. However, it is imperative to broaden the diagnostic perspective to include various other potential causes of red eyes, such as conjunctivitis, scleritis, uveitis, and episcleritis. Cognitive biases are the antithesis to accurate diagnosis, and type 1 thinking is criticised for inaccuracy in diagnosis due to its inherent vulnerability to biases [6]. The evidence, however, from experts is that the rate of diagnostic accuracy is high, with reported sensitivities close to 80% for acuity and disposition prediction [12]. Further evidence suggests that interventions to mitigate biases do not actually increase diagnostic accuracy either; rather, only interventions to address knowledge deficits do [13].

Type 2 Thinking

This slower, more nuanced model of thinking occurs when a clinician encounters a case where an obvious diagnosis is not immediately available, for example, a case of atypical optic neuritis or a case of posterior segment uveitis. Even amongst experts, a more hypothetico-deductive approach is taken in these conditions due to the nature of working through a mental checklist or differential diagnosis. This type of approach, including and excluding diagnoses, can be taxing on working memory and subsequent cognitive load [14].

Different types of memory stores

When formulating plans to help patients, clinicians often use three memory stores working synergistically: semantic, episodic, and procedural. For example, in the context of seeing a patient with a red eye, the semantic memory network draws on the general knowledge previously learnt. This includes knowing the potential causes, associated symptoms and differential diagnosis. This is used alongside the episodic memory pathway, as the clinician recalls specific instances of seeing patients with red eyes as a form of pattern recognition. Finally, as the clinician begins to examine the patient, they engage their procedural memory, particularly when using the slit lamp and recognising diagnostic signs. Where knowledge lacks in a naïve clinician, learning results and specific memory stores can be strengthened. Knowledge gaps in these memory stores separate levels of expertise. Ultimately, by engaging all three memory types, medical professionals can enhance their overall understanding and proficiency in diagnosing and treating eye-related conditions.

Cognitive load theory

The cognitive load theory by Sweller in 1988 has been a seminal change in the understanding of teaching and learning in the modern era [15]. The theory continues to inform best practices for teaching and learning and emphasises the limited capacity of working memory to retain information. This means that when teaching, limiting the amount of visual or verbal instruction required to what is salient is essential for effective learning.

This theory also applies to the process of diagnosis, where information overload can tax working memory and limit one’s ability to formulate an appropriate differential diagnosis. Understanding the limitations of working memory allows us to appreciate why novices may struggle to make a diagnosis and underpins the behavioural science behind what may be occurring in junior trainees when asked to formulate a differential diagnosis.

For example, as they are naive to using the slit lamp and recording findings, their working memory is being engaged by the time they arrive at interpreting their findings and all the previous processes have fatigued their working memory. As they gain more ability to action these processes, such as viewing the fundus or using the slit lamp, as part of their type 1 thinking system, more working memory becomes available for them to interpret and recognise their findings. When the human mind tends to solve problems in a less effortful and simpler way, this is referred to as the cognitive miser function [16]. As their development continues over time with an adequate period of rest and recovery, reinforcement of repetitive actions and the formation of new knowledge networks results in incrementally less reliance on working memory for repetitive tasks. This highlights why experts are able to ‘multi-task’ and hold multiple areas of information when compared to novices; in particular, they are already often forming a differential diagnosis list while examining and searching for specific signs to approve or disprove their expectations.

Zone of proximal development

Vygotsky’s theory regarding the zone of proximal development (ZPD) refers to the optimal environment for learners to develop their skills [17]. The gap between what they can do independently and completely unable to do lies in the ZPD, something they are able to perform with the guidance or instruction of someone with adequate knowledge or expertise. Therefore, when a trainee first learns cataract surgery, they have never made a corneal incision or inserted the lens, and support is required for each step. As more of the task enters their long-term memory (procedural in the case of practical tasks), the task falls outside the ZPD, and they then perform the next task within the ZPD, ultimately to the fellowship level where they require independent practice to remain in the ZPD. This then leads into the concept of deliberate practice. The concept of scaffolding is something surgical trainers are very familiar with. For learning to be efficient, a student-centred approach is required and the effort is collaborative rather than didactic. Therefore, a trainer provides a ‘learning scaffold’ as in instructional scaffolding theory, on which the student is provided dynamic support on which to learn [18]. This takes place by the instructor anticipating the likely errors and providing support to minimise this and then slowly removing these scaffolds as the trainee becomes more competent. For example, in cataract surgery, the trainer may fill the eye through a paracentesis with hyaluronic acid (Healon) prior to the junior making a corneal wound as emptying the anterior chamber is a common early error and then remove that step when good wound construction has been acquired. Another aspect of scaffolding is the ability to provide emotional support, manage frustration and other non-cognitive skill tasks required in learning.

Deliberate practice

Once the trainee has developed a significant level of independence, they are then required to perform deliberate practice [19]. Often trainees or indeed consultants may have achieved a level of competence where they are not required to improve further in order to discharge their clinical duties, and this model of expertise is seen as a flatlining that occurs [20]. However, some individuals who, due to performing deliberate practice, continue to become sub-experts in particular skills or fields.

Deliberate practice is defined as an effortful practice that is purposeful and systematic as opposed to repetitions that may be mindless. For example, a trainee or an expert may seek to make a more perfect capsulorrhexis, video their surgery and look for ways to improve efficiency. They may reflect on cases in the published literature, attend meetings and present cases for ongoing criticism and reflection. Amongst established consultants, this can be potentially challenging and is uncommon practice; however, to develop a sub-expertise, it is a requirement. Some sub-experts perform deliberate practice by continuing to pursue research activities and innovations within ophthalmology. The problem with deliberate practice is that one remains in the ‘learning’ phase and is required to put in consistent struggle and reflection to improve. As one becomes increasingly competent, the avenues for this become fewer, and therefore an increasing amount of effort is required. Much like in physical training, as one becomes more able, the load can either be maintained for stability or increased for growth. Although increased effort is required, there is evidence that this process of ongoing learning and the process of growth stimulates the release of dopamine. This acts as an internal positive feedback loop and drives motivation and the desire for further growth [21].

Semantic competence and problem representation

The above summarises some of the theories that impact novice learners from beginner to expert. One key component of diagnosis is the term semantic competence [11]. This is the ability to translate lay terminology from a patient’s communication into specific and precise medical terminology. For example, a patient who says they ‘cannot see in the right eye since this morning’ is translated into acute unilateral vision loss. A fluffy white patch is translated into a ‘cotton wool spot’. There is a significant correlation between semantic competence and expertise [11]. Much of this is already known, especially to experienced clinicians. A lesser known perhaps is the idea of mental representation. This is a short summary of the patient’s clinical problem and presenting concern. This is the step that should precede differential diagnosis but follow the translation of the patient’s history and examination into medical terminology. This step of mental representation allows the clinicians to recollect their findings, activate knowledge networks in long-term memory and have a greater chance of making an accurate diagnosis.

Self-regulated learning (SRL)

The previous brief explanations of the learning science theories have explored the interplay of memory and learning. Particularly from the perspective of the trainer and facilitator of learning. SRLs are an approach to learning from the student or trainee perspective. SRL is the ability of the individual to use learning strategies to achieve their learning goals [22].

SRLs are a set of demonstrable behaviours; they start with goal setting or forethought. This would equate to the personal development plans (PDPs) set at each rotation. High-performing students employ SRLs to goal set, monitor and perform metacognition (thinking about thinking), seek feedback and perform cyclical loops of the process in order to improve. High-performing students develop strategies that allow them to continuously strive in an effort to reach predetermined outcomes [22]. They have strategies in place to ensure motivation, whether intrinsic or extrinsic, consistency in effort, effective learning strategies, coping mechanisms for stress and feedback systems. Understanding how a trainee may lack or need support in either of these areas can allow trainers and trainees to engage in a process whereby the student identifies where they lack SRL and can allow for a framework within which to work.

Practical advice

Cognitive error contributes to diagnostic error significantly [6]. Therefore, it is important to understand how to minimise this from the point of teaching clinical reasoning. This may be applied to ophthalmology to reduce diagnostic errors and morbidity.

Being aware of, and applying the dual process theory [7], may enable ophthalmologists to be more cognisant when making their diagnosis. Understanding the different approaches between type 1 and type 2 thinking for both common and unusual cases, respectively, may lead to more calculated decision-making and better clinical reasoning.

Sweller's cognitive load theory [15] has allowed medical professionals to better understand and apply how to teach junior speciality trainees, who are facing more novel information that is acting as a high load on their working memory. As the trainees rely less on their working memory over time, become more assured in their practical skills and can elicit clinical signs with more confidence, they will be able to move closer to forming differential diagnoses with more ease. This can explain why specialist training year 1 (ST1) typically focuses on initially gaining core competencies specific to ophthalmology, such as slit lamp examination, applanation tonometry, and fundus examination. Once the trainee has built familiarity in these areas, and mastered these, they can then build upon their foundation of knowledge with further diagnostic skills and clinical reasoning. 

Ophthalmology trainees rely upon the teaching of surgical skills to scaffold their technique, which transforms into deliberate practice. This highlights that a learner-centred approach is highly effective in the teaching of these practical skills in order to shift out of the ZPD and into deliberate practice. As ophthalmology is a rapidly developing field, deliberate practice is required by trainees in their continual learning process, to stay relevant to changing advances in surgery and research.

Clinical supervisors in ophthalmology should be aware of the importance of the trainee demonstrating SRL, and how this may impact a trainee's success. As the trainees are at this stage, adult learners should use SRL and assume responsibility of their own knowledge. This will contribute to their lifelong learning needs, as the trainee advances from a novice learner/advanced beginner to an expert.

Understanding the principles of various teaching and learning theories will facilitate continual improvement within the ophthalmic teaching programme, leading to improved educational outcomes for trainees.

## Conclusions

The various mechanisms discussed in this article provide insight into how a clinician approaches the assessment, diagnosis, and management of a patient. A good understanding of these concepts is fundamental for ophthalmologists. It enhances their ability to provide high-quality, evidence-based care, fosters critical thinking skills, and supports ongoing professional development in the ever-evolving field of ophthalmology. This article also highlights the necessity for additional research in the field of ophthalmic education. Researchers and educators should be encouraged to evaluate the practical application and impact of these learning theories in authentic educational environments, and ultimately, to determine whether these theoretical frameworks explored can be supported by empirical data.
